# Trends in CV mortality among patients with known mental and behavioral disorders in the US between 1999 and 2020

**DOI:** 10.3389/fpsyt.2023.1255323

**Published:** 2023-11-01

**Authors:** Tanya Ebert, Nashed Hamuda, Efrat City-Elifaz, Ofer Kobo, Ariel Roguin

**Affiliations:** ^1^The Ruth and Bruce Rappaport Faculty of Medicine, Technion – Israel Institute of Technology, Haifa, Israel; ^2^Department of Psychiatry, Hillel Yaffe Medical Center, Hadera, Israel; ^3^Department of Cardiology, Hillel Yaffe Medical Center, Hadera, Israel

**Keywords:** cardiovascular mortality, mental and behavioral disorders, substance abuse, mental disorder, behavioral disorders

## Abstract

**Introduction:**

Patients with mental disorders are at increased risk of cardiovascular events. We aimed to assess the cardiovascular mortality trends over the last two decades among patients with mental and behavioral co-morbidities in the US.

**Methods:**

We performed a retrospective, observational study using the Centers for Disease Control and Prevention Wide-Ranging Online Data for Epidemiologic Research (CDC WONDER) Multiple Cause of Death dataset. We determined national trends in age-standardized mortality rates attributed to cardiovascular diseases in patients with and without mental and behavioral disorders, from 1999 to 2020, stratified by mental and behavioral disorders subtype [ICD10 codes F], age, gender, race, and place of residence.

**Results:**

Among more than 18.7 million cardiovascular deaths in the United States (US), 13.5% [2.53 million] were patients with a concomitant mental and behavioral disorder. During the study period, among patients with mental and behavioral disorders, the age-adjusted mortality rate increased by 113.9% Vs a 44.8% decline in patients with no mental disorder (both *p*_<0.05_). In patients with mental and behavioral disorders, the age-adjusted mortality rate increased more significantly among patients whose mental and behavioral disorder was secondary to substance abuse (+532.6%, *p*_<0.05_) than among those with organic mental disorders, such as dementia or delirium (+6.2%, P_−_ nonsignificant). Male patients (+163.6%) and residents of more rural areas (+128–162%) experienced a more prominent increase in age-adjusted cardiovascular mortality.

**Discussion:**

While there was an overall reduction in cardiovascular mortality in the US in the past two decades, we demonstrated an overall increase in cardiovascular mortality among patients with mental disorders.

## Highlights

- Question: To assess current cardiovascular mortality trends of patients with known mental and behavioral disorders.
- Findings: In this analysis of over 2.5 million cardiovascular deaths, we observed an increase in age-adjusted mortality rate in patients with mental and behavioral disorders. The most prominent increase was observed in substance abuse-related mortality, male patients, and residents of rural areas.
- Meaning: Our finding may indicate worsening inequity in healthcare access. Efforts must be made to ensure proper healthcare access and risk factor management of this deprived population and improve its cardiovascular health.

## Introduction

Patients with mental and behavioral disorders, including schizophrenia, bipolar disorder, major depressive disorder, and related disorders, have a low life expectancy of 7–24 years compared to the general population, because of self-harm, violence, unhealthy lifestyle habits, and physical diseases (1–4). Patients with psychiatric disorders are three-fold more likely to smoke (5) and have a higher burden of cardiometabolic risk factors including diabetes mellitus, hypertension, dyslipidemia, and obesity (6, 7), and frequently have a dual diagnosis that refers to substance use disorder (SUD) (8). Substance abuse significantly increases morbidity and mortality, especially among cocaine, heroin, and amphetamine users (8–11). During the past decade, there has been an increase in the number of patients with drug overdose who were fixated in the United States (US) (12). Methamphetamine abuse alone or with opioids together has been rapidly increasing in the United States and is currently one of the leading causes of death in the country (13). A recent study reported that both medical and external mortality increased dramatically from 1999 to 2019 in the United States among patients with substance abuse, and cardiovascular (CV) diseases were found to be the predominant medical main cause of death in this cohort (10). Many patients with substance abuse disorder have a concomitant psychiatric disorder which further increases their cardiovascular risk (14, 15).

While the overall cardiovascular mortality rate in the Western world is in decline, current data on the cardiovascular mortality rates among patients with mental and behavioral disorders is missing. Therefore, we aimed to assess the cardiovascular mortality trends over the last two decades among patients with mental co-morbidities in the US.

## Methods

In this retrospective study, the number of deaths and crude- and age-adjusted mortality rates between 1 January 1999 and 31 December 2020 were obtained from the Centers for Disease Control and Prevention Wide-Ranging Online Data for Epidemiologic Research (CDC WONDER) Multiple Cause of Death dataset. The method we used was previously described when studying a different population (16).

The Multiple Cause of Death data available on CDC WONDER are county-level national mortality and population data spanning the years 1999–2020. Data are based on death certificates for US residents. Each death certificate contains a single underlying (main) cause of death, up to 20 additional multiple causes, and demographic data. The number of deaths, crude death rates, age-adjusted death rates, and 95% confidence intervals for death rates can be obtained by cause of death (4-digit ICD-10 codes), place of residence (national, region, division, state, and county), age (single-year-of age, 5-year age groups, 10-year age groups, and infant age groups), race (American Indian or Alaskan Native, Asian/Pacific Islander, Black or African American, white), gender, and year. Data are also available for place of death, month and weekday of death, and whether an autopsy was performed.

Age-adjusted mortality rates are provided in the CDC WONDER database and are calculated using the direct method, based on data from the 2000 US census as the standard population. The underlying cause of death is the disease or injury that initiated the series of events leading directly to death. A contributing cause of death is defined as a disease or injury that can be considered a contributing factor leading to death. The underlying cause of CV death was determined using codes I00-I99 (Diseases of the circulatory system) from the International Statistical Classification of Diseases and Related Health Problems, Tenth Revision (ICD-10).

The presence of mental and behavioral disorders was determined by the presence of the codes F01-F99 (Mental and behavioral disorders) as a contributing cause of death, in the death certificate. Using 1999’s age-adjusted mortality rate as a baseline, we directly calculated the % change (% change from the baseline year) in the age-adjusted mortality rate for the study period. We analyzed trends in cardiovascular mortality of patients with mental disorders stratified by mental disorder type, age, gender, race, and place of residence (region and urbanization status). Trends were assessed based on the size of the Pearson correlation coefficient using the 20-year mortality rates. Statistical significance was set at the 0.05 level.

## Results

### Overall trends

We examined a total of 56,806,341 deaths between 1999 and 2020, of which 18,783,791 (33.1%) were defined as CV deaths. Of these, 13.5% (2.53 million) were patients with a concomitant mental and behavioral disorder (ICD-10 codes F01-F99) (Supplementary Figure S1).

**Figure 1:**
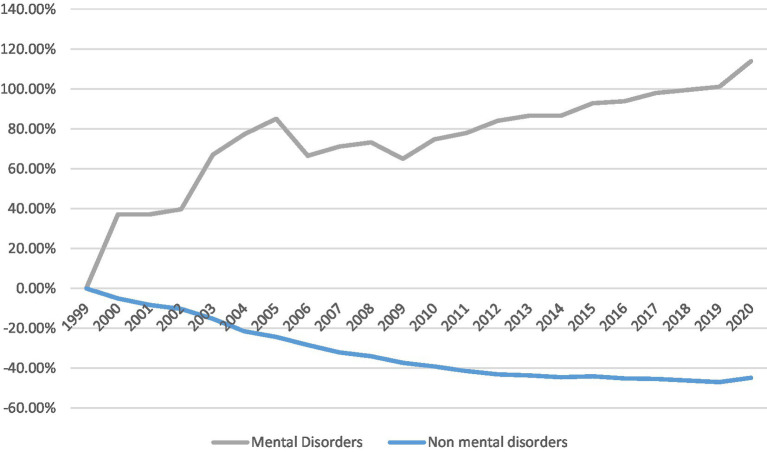
% change in age-adjusted mortality in patients with vs. without mental disorders.

The total number of CV-related deaths and crude and age-standardized CV mortality rates in the overall population and those with mental disorders are shown in Table 1. Over the last two decades, both crude and age-adjusted mortality rates have declined in patients without mental and behavioral disorders (*p*_trend_ < 0.05). In contrast, the CV mortality rate in patients with mental and behavioral disorders significantly increased (*p*_trend_ < 0.05), as shown in Figure 1.

**Table 1:** CV death among patients with and without mental and behavioral disorders.

	Total CV death			Crude CV mortality rate*			Age-adjusted CV mortality rate*		
	1999 n- 954,339	2020 n- 928,741	Percentage change	1999	2020	Percentage change	1999	2020	Percentage change
Non-mental and Behavioral disorders patients	901,620	756,303	−44.8%	323.1	229.6	−28.9%	331.4	182.9	−44.8%
Mental and behavioral disorders									
Any	52,719	172,438	+227.1%	18.9	52.3	+176.7%	19.4	41.5	+113.9%
Organic mental disorder	35,071	57,234	+63.2%	12.6	17.4%	+38.1%	13	13.8	+6.2%
Secondary to substance abuse	11,675	113,330	+870.7%	4.2	34.4	+719%	4.3	27.2	+532.6%
Mood disorders	4,595	5,196	+13.1%	1.6	1.6	0%	1.7	1.3	−23.5%

*Mortality rates are per 100,000 people in the US general population.

In 1999, out of a population of over 279 million in the US, 52,719 patients with mental and behavioral disorders experienced CV mortality, reflecting an age-adjusted mortality rate of 19.4 per 100,000. Overall, among patients without mental and behavioral disorders, 901,620 had CV mortality, an age-adjusted rate of 331.4 per 100,000.

In 2020, when the US population was over 329 million, the number of CV-related deaths increased to 172,438 (age-adjusted mortality rate of 41.5, increased by 113.9%) among patients with mental and behavioral disorders, but reduced to 756,303 (age-adjusted mortality rate of 182.9, a 44.8% reduction) among patients without mental and behavioral disorders.

### Trends by mental and behavioral disorder subtype

In 1999, among patients with mental and behavioral disorders, CV mortality was highest in those with organic mental disorders (66.5% of all mental and behavioral-related CV deaths). In 2020, CV mortality was highest in those with substance abuse (65.7% of all mental and behavioral-related CV deaths).

The age-adjusted CV mortality rate of patients with organic mental disorder increased by 6%, from 13 to 13.8 per 100,000 over 20 years (*p*_trend_- nonsignificant), while in patients with substance abuse, it significantly increased almost by 7 times, from 4.3 to 27.2 per 100,000 (*p*_trend_ < 0.05).

**Figure 2:**
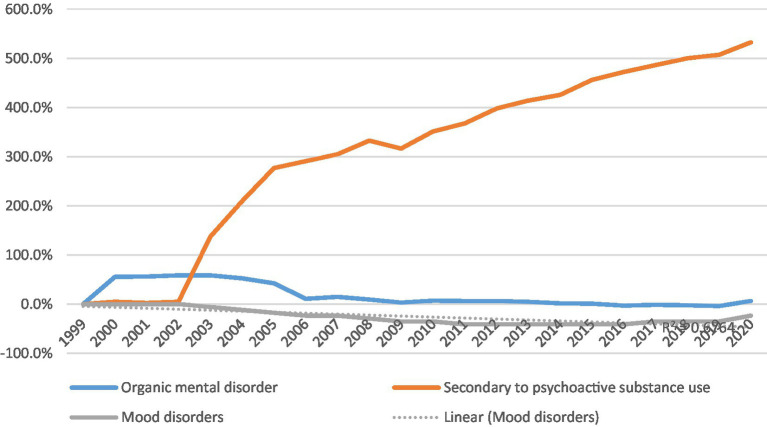
% change in age-adjusted CV mortality rate by mental disorder subtype.

In contrast, among patients with mood disorders, the age-adjusted mortality rate decreased from 1.7 to 1.3 per 100,000 population (23.5% reduction, *p*_trend_ < 0.05) (Table 1 and Figure 2).

### Trends by age, gender, and race

Men with mental and behavioral disorders experienced higher age-adjusted CV mortality than women (20.6 vs. 17.9 per 100,000 population in 1999 and 54.3 vs. 30.6 per 100,000 population in 2020). Over the study period, the increase in age-adjusted mortality was more prominent in men (163.6% vs. 70.9%; Table 2 and Supplementary Figure S2, both ptrend<0.05).

**Table 2:** CV death among patients with mental disorders.

	Total CV death			Crude CV mortality rate*			Age-adjusted CV mortality rate*		
	1999 n-52,719	2020 n-172,438	Percentage change	1999	2020	Percentage change	1999	2020	Percentage change
Gender									
Men	21117	98858	+368.1%	15.4	60.9	+295.5%	20.6	54.3	+163.6%
Women	31602	73580	+132.8%	22.2	44	+98.2%	17.9	30.6	+70.9%
Age									
Under 55	3773	13624	+261.1%	1.7	5.9	+247.1%	N/A		
55–64	3276	27178	+729.6%	13.8	64.1	+364.5%	N/A		
65–74	5728	35642	+522.2%	31.1	109.5	+252.1%	N/A		
75–84	14516	40496	+179.0%	118.7	246.2	+107.4%	N/A		
Over 85	25424	55495	+118.3%	612.0	833.5	+36.2%	N/A		
Race									
American Indian or Alaskan Native	188	1321	+602.7%	6.6	27	+309.1%	16	34.3	+114.4%
Asian or Pacific Islander	374	3103	+729.7%	3.3	13.8	+318.2%	7.4	14.4	+94.6%
Black or African American	5245	21217	+304.5%	14.5	45	+210.3%	22.7	49.4	+117.6%
White	46912	146797	+212.9%	20.5	57.6	+181.0%	19.3	42.1	+118.1%
Region									
Northeast	9614	29004	+201.7%	18	51.9	+188.3%	16.5	37.4	+126.7%
Midwest	13440	44365	+230.1%	21	64.9	+209.0%	20.3	49.8	+145.3%
South	20038	67789	+238.3%	20.2	53.5	+164.9%	21.3	34.5	+62.0%
West	9627	31280	+224.9%	15.4	39.8	+158.4%	18.1	33.5	+85.1%
Urbanization									
Large central metro	13408	39385	+193.7%	15.6	38.9	+149.4%	17.6	34.2	+94.3%
Large fringe metro	11069	38206	+245.2%	16.8	46	+173.8%	18.5	36.9	+99.5%
Medium metro	11331	38772	+242.2%	19.9	56	+181.4%	20.3	43.6	+114.8%
Small metro	5679	19687	+246.7%	22	65.7	+198.6%	21.5	49.1	+128.4%
Micropolitan (nonmetro)	6251	20518	+228.2%	24.2	75.2	+210.7%	21.9	54.1	+147.0%
Noncore (nonmetro)	4981	15870	+218.6%	26.5	84.8	+220.0%	21	55.1	+162.4%

*Mortality rates are per 100,000 people in the US general population.

Over the study period, we observed an increase in crude CV mortality rates amongst mental and behavioral patients across all age groups (all *p*_trend_ < 0.05). The magnitude of increase was higher among patients in the 55–64 years of age group as compared with other age groups (364.5% increase in the 55–64 age group vs. 36.2–252.1%, Table 2, and Supplementary Figure S3).

Compared with other ethnic groups, Black or African American patients with mental and behavioral disorders experienced the highest age-adjusted CV mortality rates (22.7 in 1999 and 49.4 per 100,000 population in 2020). The greatest increase in age-adjusted CV mortality rate was observed in white patients, while the Asian or Pacific Islander patients had the lowest increase (118.1% vs. 94.6% respectively, *p*_trend_ < 0.05) (Table 2 and Supplementary Figure S4).

### Trends by geography

In 1999, the age-adjusted CV mortality was similar in mental and behavioral disorders patients located in metro and non-metro areas (17.6–21.5 per 100000 population and 21–21.9 per 100000 population, respectively). During the study period, the increase in CV mortality was more prominent in the non-metro areas, leading to higher age-adjusted CV mortality in the non-metro areas than in metro areas [54.1–55.1 vs. 34.2–49.1, all (*p*_trend_ < 0.05) per 100,000 population].

The greatest increase in age-adjusted CV mortality in patients with mental and behavioral disorders was observed in the noncore (non-metro) area, and the lowest increase was observed in the large central metro area (162.4% vs. 94.3% respectively) (Table 2 and Supplementary Figures S3, S5).

## Discussion

**Figure 3:**
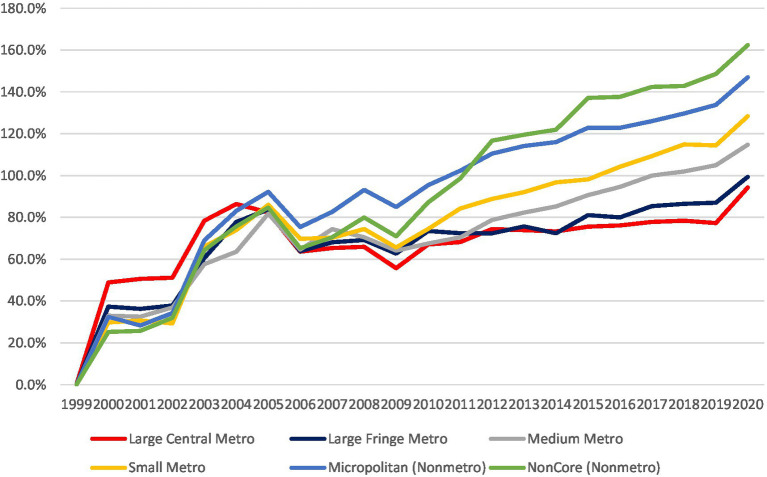
% change in age-adjusted mortality by urbanization status.

Our findings demonstrate that the overall temporal decrease in cardiovascular mortality in the US over the last 20 years, was not observed among patients with mental and behavioral disorders, and specifically those with substance abuse. Among patients with mental and behavioral disorders, we observed a significant increase in cardiovascular mortality, mainly among males, the white population, those aged 55–64 years, and residents of rural areas. During the study period, the male–female and rural–urban cardiovascular mortality gaps increased among patients with mental and behavioral disorders (Figure 3).

It has been previously reported that the increase in CV mortality among patients with mental and behavioral disorders may be related to a higher prevalence of CV risk factors among these patients (17, 18). Several explanations have been proposed for the excess mortality in these groups: side-effects of pharmacological treatment (19), unhealthy diets (20), high prevalence of cigarette smoking, and limited physical activity (21). Furthermore, patients with mental and behavioral disorders generally have reduced access to healthcare. This is both due to their socioeconomic conditions as well as negative attitudes from clinicians working outside the mental health fields toward patients with severe mental illness, and the negative social consequences of having a mental disorder (22–24).

It is known that patients with mental and behavioral disorders frequently have a dual diagnosis that refers to substance use disorder (25–32). Approximately half of the patients with schizophrenia have a lifetime diagnosis of SUD (33). According to the literature, traditionally most CV mortality is caused by cocaine, heroin, and amphetamine use (8–11). However, opioid overdose contributed to the decrease in the life expectancy of Americans from 78.8 to 78.5 between 2014 and 2017. Unfortunately, the number of patients who use substances has increased and it has caused an increase in CV mortality rate in these people. Opioid abuse was more common among white patients, males, and patients who were 45–55 years of age and this may contribute to the significant increase in CV mortality among white men that was found in our population with mental disorders (34). Another possible explanation for lower CV mortality in women compared to men is that primary prevention strategies, such as the adoption of several healthy lifestyle behaviors and the use of proven medicines, are generally more prevalent in women than men, but this study did not examine a specific population with mental and behavioral disorders or SUD (35). However, it is important to note that, in our study, the number of women who died due to CV events and had comorbid mental and behavioral disorders was three times lower than the men.

In general, despite the prominent increase in mortality among white patients, we observed higher mortality rates during the entire study period among Black patients. This finding is primarily explained by racial differences in socioeconomic status, CV disease risk factors, higher proportions of comorbidities, and reduced access to healthcare (36). In a recent study, similar results were found, which showed an increase in CV mortality in Black patients that was attributable to socioeconomic factors and a high prevalence of lifestyle, psychosocial, and clinical risk factors (37).

In our study, we found that the age-adjusted CV mortality rate dropped by 23.5% among patients with mood disorders. This may be a result of successful campaigns as there has been an increase in awareness and improvements in the diagnosis and treatment of mood disorders among primary care physicians over the last decades. The overall access to treatment of patients with mood disorders has improved significantly and physician prejudice toward these patients has decreased. Increased accessibility to antidepressant treatment may have ultimately led to a decrease in CV morbidity and mortality among older men with depression (38).

We observed a significantly more prominent increase in CV mortality of patients with mental and behavioral disorders in rural areas compared to metro areas. Access to healthcare providers varies in the US, and residents of rural areas may have less access to specialty care. The management of cardiovascular disease in patients with mental and behavioral disorders requires expertise that may be difficult to access in rural communities. Indeed, there has been a disproportionate closure of hospitals in rural areas (39). Rural residents, specifically those with mental and behavioral disorders, are more likely to be affected by social determinants of health that impact cardiovascular outcomes, such as lower household incomes, lower food security, lower educational attainment, higher rates of being uninsured, and transportation barriers. Comorbid conditions such as diabetes, hypertension, and obesity are also more common in rural areas (40).

Further prospective longitudinal studies on a large cohort of patients with mental and behavioral disorders may be needed to verify our conclusion and to examine factors contributing to CV mortality reduction in this cohort.

## Limitations

This study has several limitations. First, because the information was obtained from an administrative database of death certificate data, there is an element of misclassification bias, particularly given the potential for inaccuracies in coding the cause of death in death certificate data. Second, the CDC WONDER database does not provide information on important contributory factors to cardiovascular mortality, such as type 2 diabetes, hypertension, and obesity. It was impossible to accurately describe the differences between cardiovascular risk factor burden amongst patients with different types of mental or behavioral disorders, prevalent cardiovascular disease and its treatment, socioeconomic status, and healthcare access. Third, there may be underdiagnoses or underreporting of mental and behavioral disorders in the death certificates. However, it is possible that in most cases where an individual with a mental disorder dies of a cardiovascular cause, the coroner will either not be aware of the mental condition or will not code it as directly contributing to the death, except for the drug abuse conditions.

## Conclusion

While there was an overall reduction in cardiovascular mortality in the US in the past two decades, we demonstrated an overall increase in cardiovascular mortality among patients with mental and behavioral disorders. The increase was more significant among patients with substance abuse-related mental and behavioral disorders, in men, and especially among residents of rural areas. Efforts must be made to ensure proper healthcare access and risk factor management of this deprived population and improve its cardiovascular health.

## Data availability statement

The original contributions presented in the study are included in the article/Supplementary material, further inquiries can be directed to the corresponding author.

## Author contributions

TE: Conceptualization, Funding acquisition, Investigation, Writing – original draft, Writing – review & editing. NH: Conceptualization, Formal analysis, Methodology, Writing – original draft. EC-E: Supervision, Writing – review & editing. OK: Conceptualization, Data curation, Formal analysis, Methodology, Writing – original draft, Writing – review & editing. AR: Conceptualization, Data curation, Investigation, Supervision, Writing – review & editing.
