# Supplementary material for: Trends in CV mortality among patients with known mental and behavioral disorders in the US between 1999 and 2020
Source: Front Psychiatry. 2023 Nov 1;14:1255323. doi: 10.3389/fpsyt.2023.1255323 (PMC10646424; doi:10.3389/fpsyt.2023.1255323)
Supplement: Supplementary file 1 [file Data_Sheet_1.docx]

Figure S1: Flow diagram of study population

All records taken from Centers for Disease Control and Prevention Wide-Ranging Online Data

56,806,341 deaths certificates

between 1999-2020

18,783,791 CV mortality

2,544,202 With mental and

Behavioral disorder

16,239,589 No mental and

Behavioral disorder

Figure S2: % change in age-adjusted mortality by gender

Figure S3: % change in crude mortality rate by age

Figure S4: % change in age-adjusted mortality by race

Figure S5: % change in age-adjusted mortality by region
